# Impact of human leukocyte antigen mismatch between donor-recipient on acute rejection in liver transplantation using next-generation sequencing: a single-center study

**DOI:** 10.3389/fimmu.2025.1576815

**Published:** 2025-05-20

**Authors:** Genjie Lu, Yangfang Lu, Yanmin He, Wei Chen, Faming Zhu

**Affiliations:** ^1^ Department of Blood Transfusion, Ningbo Medical Center Lihuili Hospital, Ningbo University, Ningbo, China; ^2^ Department of Radiotherapy, Ningbo Medical Center Lihuili Hospital, Ningbo University, Ningbo, China; ^3^ HLA Typing Laboratory, Blood Center of Zhejiang Province, Hangzhou, China

**Keywords:** liver transplantation, acute rejection, human leukocyte antigen, mismatch, next-generation sequencing

## Abstract

**Background:**

The effect of human leukocyte antigen (HLA) mismatch on acute rejection (AR) in liver transplantation (LT) is controversial. This study aimed to investigate the effect of donor-recipient mismatch at the HLA-A, -B, -C, -DRB1, -DRB3, -DRB4, -DRB5, -DQA1, -DQB1, -DPA1, and -DPB1 loci on AR in LT.

**Methods:**

In total, 92 patients who underwent LT were selected for investigation from 1 January 2018 to 30 June 2024, and the donors of these patients were also from the same hospital. All donor and recipient specimens were genotyped via next-generation sequencing (NGS) for the 11 HLA loci. The patients were divided into AR and non-AR groups according to whether AR occurred after LT.

**Results:**

A total of 12 cases (13.04%) experienced AR after LT. The proportion of chronic hepatitis B virus (HBV) infection was lower in the AR group than that in the non-AR group (*P*<0.05), while the proportion of split LT and mortality within 1 year after transplantation was higher in the AR group than in the non-AR group (*P*<0.05). Compared with the non-AR group, the AR group had a significantly higher proportion of high-mismatch DQB1 (2 vs. 0-1) and DRB1+DQB1 (4 vs. 0-3) (*P*<0.05) at the allele level, and other mismatches of a single locus and different combinations of the 11 HLA loci had no significant differences between the two groups (*P*>0.05). However, neither high-mismatch DQB1 nor high-mismatch DRB1+DQB1 at the allele level was an independent risk factor for AR after adjustment for chronic HBV infection, LT operative procedures, and immunosuppressive regimen using bootstrapping [odds ratio (OR): 0.203, 95% confidence interval (CI): 0.000–1.300, *P*=0.067; OR: 0.404, 95% CI: 0.000–2.625, *P*=0.172, respectively].

**Conclusion:**

In this preliminary study, no correlation between HLA mismatch at the allele level and post-transplant AR episodes was found.

## 1 Introduction

Over the past 50 years, liver transplantation (LT) has become common in many countries worldwide (1). Up to now, LT is still the only curative treatment for end-stage liver disease (ESLD) (2). Indications for LT include cirrhosis caused by chronic viral infection, excessive alcohol consumption, metabolic dysfunction-associated steatotic liver disease (MASLD), and liver cancer or acute liver failure (3). In recent years, many research studies focused on LT effects have emerged, including the source of the donor liver (4–6), prognosis (7, 8), and acute rejection (AR) (9, 10).

AR is a common complication after LT, usually within the first 3 months after surgery (2). AR can be divided into acute cellular rejection (ACR) and antibody-mediated rejection (AMR) (11). However, AMR is very rare in LT (2). AR after LT is mainly mediated by T cells, with an incidence of approximately 10%–35% (12–14). Studies have reported that AR may be associated with a variety of factors, including ABO blood group incompatibility (14), hepatitis B virus (HBV) infection (15), and human leukocyte antigen (HLA) mismatch (16, 17).

HLA molecules are expressed on the surface of a variety of cells. The HLA region is one of the most polymorphic genes in the human genome. As of January 2025, 41,003 HLA alleles have been identified in the latest IPD-IMGT/HLA database version 3.59.0 (18). There are significant differences in the distribution of HLA loci among different populations and ethnic groups (19, 20). It has been proven that HLA plays a vital role in transplantation immunity, including hematopoietic stem cell transplantation (HSCT) (21) and organ transplantation (22, 23). However, the association between donor-recipient HLA mismatch and AR after LT is inconsistent (16, 17).

Therefore, this prospective cohort study was conducted to understand better the association between donor-recipient HLA mismatch and AR in LT in the Chinese population in the Ningbo region, Zhejiang Province, China.

## 2 Materials and methods

### 2.1 Study design, population, and specimen

**Figure 1:**
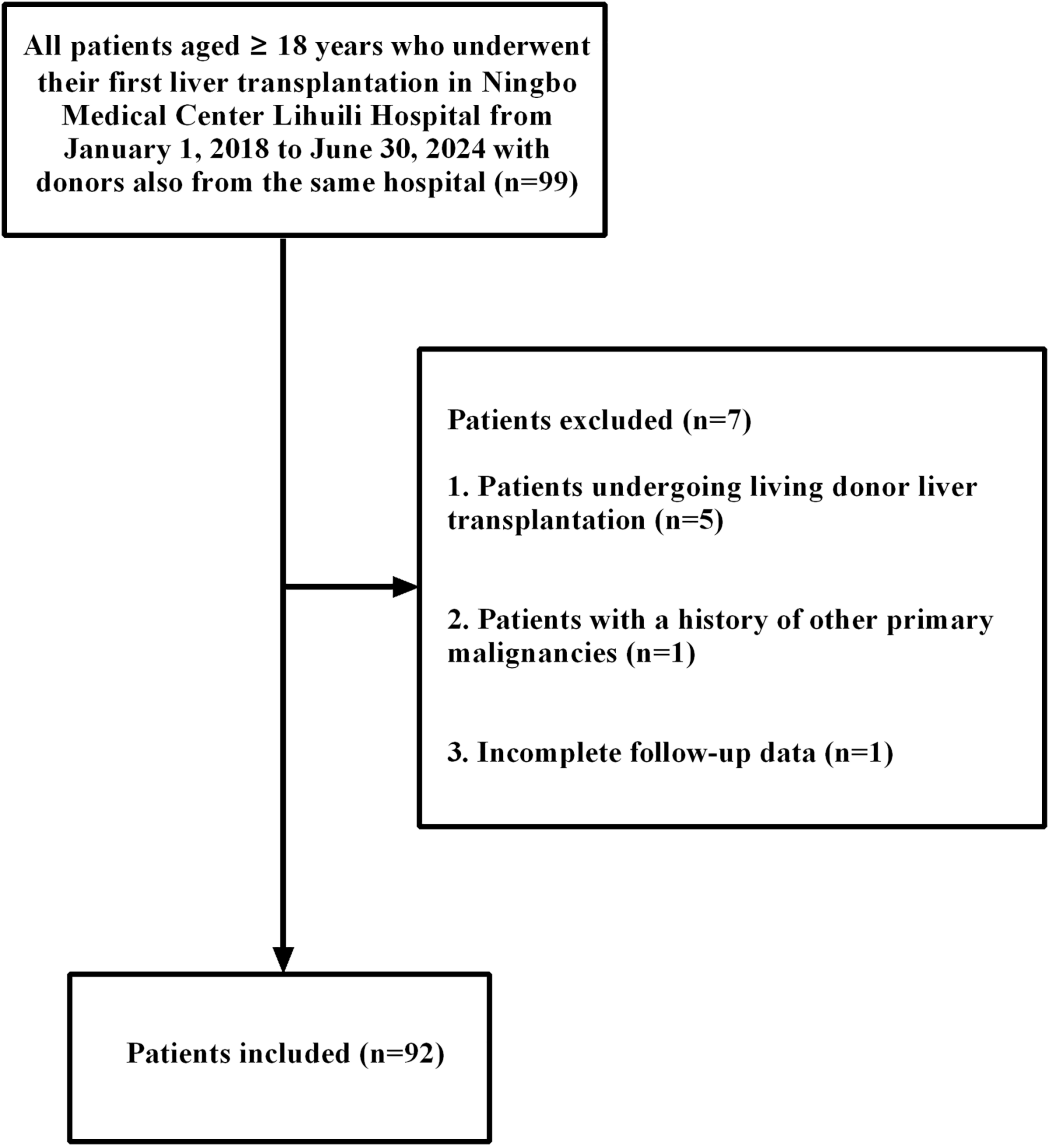
Flow diagram of the study.

All patients who underwent LT in Ningbo Medical Center Lihuili Hospital and whose donors were also from the same hospital were selected for investigation from 1 January 2018 to 30 June 2024. LT was performed according to either the Milan criteria (24) or the Hangzhou criteria (China) (25). Liver transplant waiting list prioritization was determined by the Model for End-Stage Liver Disease (MELD) score, which incorporated special exceptions for patients with hepatocellular carcinoma (HCC). All donor organs were allocated fairly and transparently through the China Organ Transplant Response System (COTRS). The inclusion criteria were as follows: (1) all donors and recipients were ≥ 18 years old; (2) all donors and recipients were from the Zhejiang Han population; (3) all patients were liver transplant recipients; (4) the donor was also from the same hospital. The exclusion criteria were as follows: (1) a history of other primary malignancies; (2) living donor liver transplantation (LDLT); (3) incomplete data. According to the inclusion criteria, 92 liver transplant patients were ultimately enrolled in this study (**Figure 1**). This study was approved by the ethics committee of Ningbo Medical Center Lihuili Hospital (Approval No: 2024-009). Written informed consent was obtained from all participants. The remaining ethylene diamine tetraacetic acid (EDTA) anticoagulant specimens for the liver transplant donors and recipients for ABO blood group testing were collected and stored below -20°C until processing.

### 2.2 Genomic DNA extraction

According to the manufacturer’s instruction, genomic DNA was extracted using a commercially available DNA extraction kit (Roche Diagnostics, Indianapolis, IN, USA). The final DNA concentration was adjusted to 20 to 30ng/µl with a purity (A260/A280) of 1.60 to 1.80.

### 2.3 HLA genotyping

As described in our previous reports in detail (20, 26), all specimens were genotyped for 11 HLA loci (HLA-A, -B, -C, -DRB1, -DRB3, -DRB4, -DRB5, -DQA1, -DQB1, -DPA1, and -DPB1) using an AllType™ next-generation sequencing (NGS) kit (One Lambda Inc, Canoga Park, CA, USA) according to the manufacturer’s instructions. At first, the full-length genomic sequences of HLA-A, -B, -C, -DQA1, and -DPA1 loci were amplified, along with the sequencing regions spanning 2 exons through the 3’ untranslated region (UTR) for HLA-DRB1/3/4/5, -DQB1, and -DPB1 loci, using a single multiplex PCR method. Following amplification, adapter ligation was performed prior to DNA fragmentation. Size selection was then conducted using magnetic beads to isolate fragments ranging from 300 to 1,000 bp, which were subsequently pooled. For library preparation, we employed the Ion Shear Plus Reagents Kit and Ion Plus Fragment Library Kit (One Lambda Inc, Canoga Park, CA). All purification steps, including amplicon purification, dilution, and final library pooling, were automated using the Microlab STAR robotic platform (Hamilton, Bonaduz, Switzerland). Final sequencing was carried out on the Ion Torrent S5 platform (Thermo Fisher Scientific, Waltham, MA, USA). HLA genotype was assigned using the Type Stream Visual Software version 2.0 (One Lambda Inc, Canoga Park, CA, USA). The ambiguous HLA allele combinations were assigned according to the Chinese common and well-documented (CWD) HLA allele catalog (27).

### 2.4 Data sources and measurement

The demographic and clinical characteristics of liver transplant donors and recipients, including age, gender, ABO blood group, body mass index (BMI), surgical method, MELD score, Child–Pugh stage, AR, and medical history, were obtained through the electronic medical record (EMR) in the hospital. The diagnosis of AR was based on histological examination after liver biopsy by professional pathologists, and the Banff rejection activity index (28). The study exclusively monitored AR episodes occurring within the first 3 postoperative months. The mortality within 1 year after LT was obtained through outpatient, inpatient, or telephone follow-up, and the follow-up time was up to 31 December 2024.

### 2.5 HLA mismatch calculation

HLA mismatch was defined as the presence of non-identical HLA alleles between the donor and recipient at a high resolution (two fields, e.g., A*02:01 vs. A*02:05). A complete match was defined as being identical in both alleles at each locus. Theoretically, there may be 0 to 2 mismatches in each HLA locus at the allele level, except for the HLA-DRB3, -DRB4, and -DRB5 loci in the donor and recipient of LT. In this study, a mismatch number of 2 vs. 0 or 1 was used to analyze the mismatch of a single HLA locus in liver transplant donors and recipients, except for HLA-DRB3, -DRB4, -DRB5 loci, for which a mismatch number of 1–2 vs. 0 was used because of the large number of blanks. In addition, a supplementary analysis referring to the study by Tajima et al. (29) was performed, which defined HLA mismatch at the allele level as the number of donor HLA loci that the liver transplant recipient did not have.

### 2.6 Immunosuppressive regimen

Anti-interleukin (IL)-2 receptor antagonists (mainly basiliximab) were used for immune induction half an hour before LT in most patients. Intraoperative immunosuppression was performed using intravenous methylprednisolone. Different combinations of calcineurin inhibitors (CNIs) (mainly tacrolimus), antimetabolic drugs [mainly mycophenolate mofetil (MMF)], mammalian target of rapamycin (mTOR, sirolimus), and glucocorticoids (methylprednisolone) were used for postoperative immunosuppression. Tacrolimus plus MMF was the most commonly used standard dual therapy. Postoperative immunosuppression was only observed at the onset of AR or at 3 months after surgery (without AR). Detailed immunosuppressive regimens were provided in **Supplementary Table S1**.

### 2.7 Treatment of AR

Patients presenting with AR episodes received dose-adjusted methylprednisolone as the first-line therapy. For those intolerant to cyclosporine, tacrolimus was substituted as an alternative immunosuppressant. In cases of steroid-resistant rejection, antilymphocyte globulin (ALG) or antithymocyte globulin (ATG) was administered. Patients who responded to treatment and had normal liver function tests were considered to have achieved resolution of AR.

### 2.8 Statistical analysis

Categorical variables were expressed as counts and percentages, and their significance was assessed using the chi-square test or Fisher’s exact test. Continuous variables conforming to the normal distribution were described as mean ± SD, and their significance was evaluated using the t-test. Continuous variables with non-normal distributions were presented as medians with interquartile ranges, and their significance was assessed using the Mann–Whitney U test. The Hardy–Weinberg equilibrium for each HLA locus was analyzed using Arlequin software 3.5.2.2 (30). Kaplan–Meier analysis was performed to plot survival curves, and intergroup differences were assessed via log-rank tests. Univariate and multivariate logistic regression analysis of each variable was performed with AR (yes or no) as the dependent variable. A *P*-value or corrected *P* (*Pc*) value less than 0.05 was considered statistically significant. All statistical calculations were performed using SPSS statistical software version 24.0 (IBM, Armonk, NY, USA), GraphPad PRISM 6.0 software (GraphPad Software, San Diego, CA, USA), or Arlequin software 3.5.2.2 (30).

## 3 Results

### 3.1 Higher death rate within 1 year after LT in the AR group than in the non-AR group

**Figure 2:**
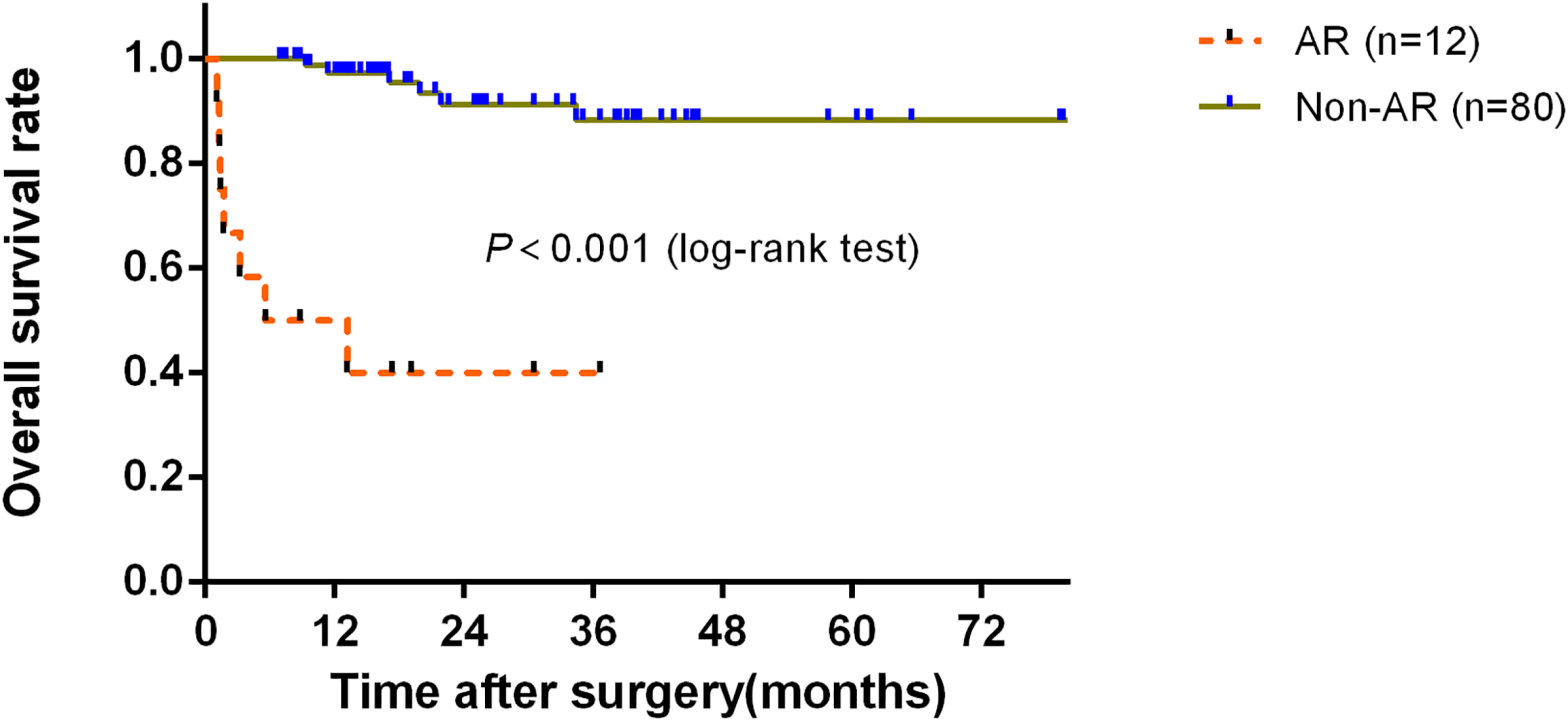
Comparison of overall survival (OS) between the AR and non-AR groups.

The clinical and demographic characteristics of 92 liver transplant patients are listed in **Table 1**. Of these transplants, 83 (90.2%) had the same ABO blood group as the donor and the others were compatible. Furthermore, 65 (70.7%) and 51 (55.4%) had chronic HBV and liver cancer, respectively. A total of 12 cases (13.0%) experienced AR. Up to the end of follow-up, 8 patients (8.7%) died 1 year after LT. Of the 8 deaths, the predominant cause was severe infection complicated by multiple organ failure (62.5%, 5/8 cases), with the remaining three cases attributed to heart failure, tumor recurrence and metastasis, and severe infection associated with gastrointestinal bleeding, respectively. **Figure 2** shows that the AR group had significantly worse 1-year overall survival (OS) than that in the non-AR group (*P* < 0.001). The differences in the median age, gender, O blood group, and liver cancer were not significant between these groups. However, the proportion of chronic HBV infection was lower in the AR group than in the non-AR group (*P*<0.05), while the proportion of split LT and mortality within 1 year after transplantation was higher in the AR group than in the non-AR group (*P*<0.001).

**Table 1:** Clinical and demographic characteristics of patients* who underwent a liver transplant.

Variable	Total (n=92)	AR (n=12)	Non-AR (n=80)	*P*-value
Median age (IQR), years	52.0 (46.5, 59.5)	56.0 (53.5, 65.0)	51.5 (46.0, 59.0)	0.058
Age > 60 years old, n (%)	21 (22.8)	4 (33.3)	17 (21.3)	0.575
Male, n (%)	68 (73.9)	6 (50.0)	62 (77.5)	0.095
Body mass index, kg/m^2^	22.40 ± 3.40	21.91 ± 2.46	22.48 ± 3.53	0.594
O blood group, n (%)	31 (33.7)	5 (41.7)	26 (32.5)	0.765
Chronic hepatitis B virus infection, n (%)	65 (70.7)	5 (41.7)	60 (75.0)	0.043
Liver cancer, n (%)	51 (55.4)	5 (41.7)	46 (57.5)	0.303
Death within 1 year after liver transplantation, n (%)	8 (8.7)	6 (50.0)	2 (2.5)	<0.001
Operative procedures of liver transplantation, n (%)				0.001
Orthotopic	49 (53.3)	1 (8.3)	48 (60.0)	
Split	43 (46.7)	11 (91.7)	32 (40.0)	
The same ABO blood group, n (%)	83 (90.2)	11 (91.7)	72 (90.0)	>0.999
Model for End-Stage Liver Disease score	22.00 (14.61, 23.70)	22.00 (17.32, 22.22)	22.00 (14.08, 23.95)	0.995
Child–Pugh stage				0.794
A	28 (30.4)	3 (25.0)	25 (31.3)	
B	27 (29.3)	3 (25.0)	24 (30.0)	
C	37 (40.2)	6 (50.0)	31 (38.8)	
Immunosuppressive regimen, n (%)				0.497
AMTM	57 (62.0)	9 (75.0)	48 (60.0)	
Others	35 (38.0)	3 (25.0)	32 (40.0)	
Hypertension, n (%)	14 (15.2)	3 (25.0)	11 (13.8)	0.561
Diabetes, n (%)	15 (16.3)	4 (33.3)	11 (13.8)	0.196

AMTM: Anti-IL-2 receptor antagonists (preoperative) + methylprednisolone (intraoperative) + tacrolimus + MMF (postoperative).

* Data are shown as n (%) for categorical variables and as median (interquartile range) or mean ± SD for continuous variables.

### 3.2 No difference in the donor characteristics in the AR group and non-AR group

The demographic and ABO blood group distribution characteristics of the 92 matched liver transplant donors are shown in **Table 2**. The median age was 47.0 years old and 5 donors (5.4%) were over 60 years old. There were 75 (81.5%) male donors and 37 (40.2%) O blood group donors. Because of the split LT, only 71 independent donors were actually available; that is, 21 donors were paired with two liver transplant recipients at the same time. The difference in the median age, gender, and O blood group was not significant between these groups.

**Table 2:** Basic demographic and ABO blood group distribution characteristics of liver transplant donors*.

Variable	Total (n=92)	AR (n=12)	Non-AR (n=80)	*P*-value
Median age (IQR), years	47.0 (35.0, 53.0)	47.5 (44.0, 56.5)	46.5 (34.0, 52.5)	0.291
Age > 60 years old, n (%)	5 (5.4)	0 (0.0)	5 (6.3)	>0.999
Male, n (%)	75 (81.5)	11 (91.7)	64 (80.0)	0.567
O blood group, n (%)	37 (40.2)	6 (50.0)	31 (38.8)	0.670

* Data are shown as n (%) for categorical variables and median (interquartile range) for continuous variables.

### 3.3 High-mismatch in the DQB1 and DRB1+DQB1 loci may be associated with AR

**Figure 3:**
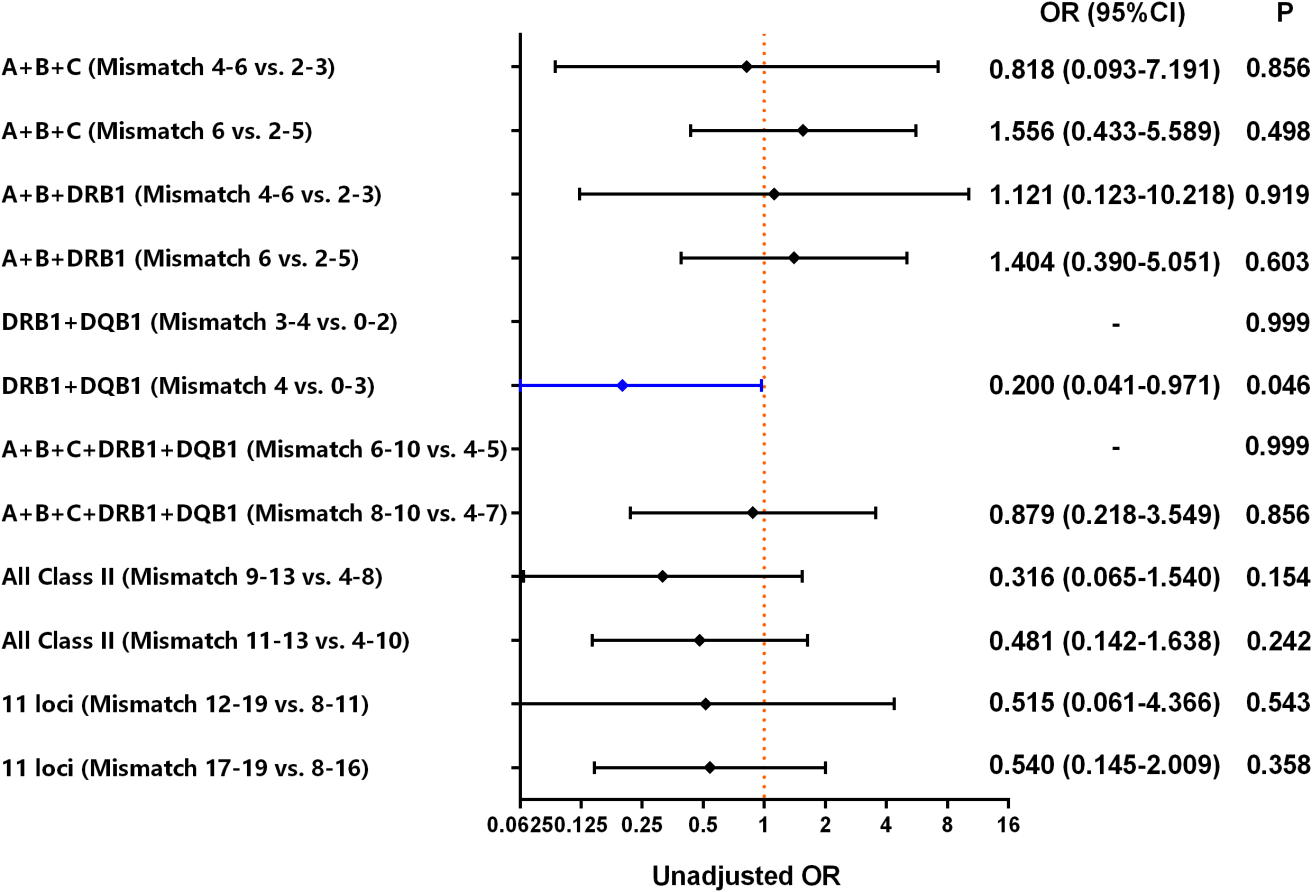
Risk of AR in liver transplant patients, according to donor-recipient mismatch between various combinations of 11 HLA loci. Class II includes HLA-DRB1, -DRB3, -DRB4, -DRB5, -DQA1, -DQB1, -DPA1, and -DPB1. The 11 loci include HLA-A, -B, -C, -DRB1, -DRB3, -DRB4, -DRB5, -DQA1, -DQB1, -DPA1, and -DPB1.

Hardy–Weinberg equilibrium was fitted in the 11 HLA loci between the liver transplant recipients and donors (**Supplementary Table S2**). However, the number of HLA mismatches at the allele level, including the mismatches of a single locus and various combinations of 11 HLA loci, was compared between the AR and non-AR groups (**Table 3**). Compared with the non-AR group, the AR group had a significantly higher proportion of high-mismatch DQB1 (2 vs. 0-1) and DRB1+DQB1 (4 vs. 0-3) (*P*<0.05). However, there were no significant differences in mismatches of other HLA loci at the allele level between the two groups (*P*>0.05) (**Table 3**, **Supplementary Table S3**, **Figure 3**). In addition, comparisons of the mismatch of a single locus and various combinations of the 11 HLA loci at the allele level between the AR and non-AR groups are provided in **Supplementary Table S4** in detail.

**Table 3:** Comparison of the number of HLA mismatches between the AR and non-AR groups.

HLA loci	Number of mismatches	AR (n=12)	Non-AR (n=80)	*P*-value
A	0-1	7 (58.3)	30 (37.5)	0.170
	2	5 (41.7)	50 (62.5)	
B	0–1	2 (16.9)	19 (23.8)	0.860
	2	10 (83.3)	61 (76.3)	
C	0–1	6 (50.0)	21 (26.3)	0.179
	2	6 (50.0)	59 (73.8)	
DRB1	0–1	2 (16.7)	20 (25.0)	0.789
	2	10 (83.3)	60 (75.0)	
DRB3	0	4 (33.3)	17 (21.3)	0.575
	1–2	8 (66.7)	63 (78.8)	
DRB4	0	4 (33.3)	41 (51.3)	0.247
	1–2	8 (66.7)	39 (48.8)	
DRB5	0	8 (66.7)	44 (55.0)	0.447
	1–2	4 (33.3)	36 (45.0)	
DQA1	0–1	2 (16.7)	23 (28.8)	0.596
	2	10 (83.3)	57 (71.3)	
DQB1	0–1	1 (8.3)	38 (47.5)	0.010
	2	11 (91.7)	42 (52.5)	
DPA1	0–1	6 (50.0)	58 (72.5)	0.214
	2	6 (50.0)	22 (27.5)	
DPB1	0–1	6 (50.0)	42 (52.5)	0.872
	2	6 (50.0)	38 (47.5)	
A+B+C	2–3	1 (8.3)	8 (10.0)	>0.999
	4–6	11 (91.7)	72 (90.0)	
A+B+C	2–5	8 (66.7)	45 (56.3)	0.496
	6	4 (33.3)	35 (43.8)	
A+B+DRB1	2–3	1 (8.3)	6 (7.5)	>0.999
	4–6	11 (91.7)	74 (92.5)	
A+B+DRB1	2–5	8 (66.7)	47 (58.8)	0.837
	6	4 (33.3)	33 (41.3)	
DRB1+DQB1	0–2	0 (0.0)	3 (3.8)	>0.999
	3–4	12 (100.0)	77 (96.3)	
DRB1+DQB1	0–3	2 (16.7)	40 (50.0)	0.031
	4	10 (83.3)	40 (50.0)	
A+B+C+DRB1+DQB1	4–5	0 (0.0)	5 (6.3)	>0.999
	6–10	12 (100.0)	75 (93.8)	
A+B+C+DRB1+DQB1	4–7	3 (25.0)	22 (27.5)	0.856
	8–10	9 (75.0)	58 (72.5)	
All Class II	4–8	2 (16.7)	31 (38.8)	0.244
	9–13	10 (83.3)	49 (61.3)	
All Class II	4–10	6 (50.0)	54 (67.5)	0.389
	11–13	6 (50.0)	26 (32.5)	
11 loci	8–11	1 (8.3)	12 (15.0)	0.862
	12–19	11 (91.7)	68 (85.0)	
11 loci	8–16	8 (66.7)	63 (78.8)	0.575
	17–19	4 (33.3)	17 (21.3)	

Class II includes HLA-DRB1, -DRB3, -DRB4, -DRB5, -DQA1, -DQB1, -DPA1, and -DPB1. The 11 loci include HLA-A, -B, -C, -DRB1, -DRB3, -DRB4, -DRB5, -DQA1, -DQB1, -DPA1, and - DPB1.

The subgroup analysis of 11 AR patients and 42 non-AR patients with donor-recipient biallelic DQB1 mismatches revealed no predominant allele-specific mismatch patterns (**Supplementary Tables S5**, **S6**). Comparative analysis of DQB1 allele frequencies was performed among 11 AR cases, 42 non-AR cases, and local healthy controls (20), including donor-to-donor, recipient-to-recipient, donor-to-healthy control, and recipient-to-healthy control comparisons (**Supplementary Tables S7**-**S12**). The results showed no statistically significant differences between the AR and non-AR groups (*Pc*>0.05). However, the frequency of DQB1*06:01 in the AR donors was significantly higher than that in the healthy controls (*Pc*=0.002), while DQB1*05:03 in the non-AR recipients was elevated compared to the controls (*Pc*=0.010).

HLA mismatch at the allele level, i.e., the number of donor HLA loci that the recipient did not have, was also analyzed (**Supplementary Table S13**). However, none of the HLA mismatches in this analysis were statistically different between the AR and non-AR groups (*P*>0.05).

### 3.4 HLA mismatch was not an independent risk factor for AR in the multivariate analysis

Binary logistic regression analysis was performed with AR as the dependent variable and the preoperative, intraoperative, donor, and HLA mismatch indexes with statistically significant differences between the AR and non-AR groups as independent variables. It was found that neither donor-recipient high-mismatch DQB1 nor high-mismatch DRB1+DQB1 was an independent risk factor for AR after adjustment for chronic HBV infection, LT operative procedures, and immunosuppressive regimen using bootstrapping [odds ratio (OR): 0.203, 95% confidence interval (CI): 0.000–1.300, *P*=0.067; OR: 0.404, 95% CI: 0.000–2.625, *P*=0.172, respectively] (**Tables 4**, **5**).

**Table 4:** Univariate and multivariate analysis of HLA-DQB1 mismatch number and other factors influencing AR risk in patients who underwent LT using bootstrapping.

Variable	Univariate analysis			Multivariate analysis		
	OR	95% CI	*P*-value	OR	95% CI	*P*-value
Chronic hepatitis B virus infection (Yes vs. NO)	4.200	1.078, 19.708	0.018	3.013	0.489, 27.938	0.169
Operative procedures of liver transplantation (split vs. orthotopic)	0.061	0.000, 0.287	0.013	0.101	0.000, 0.486	0.011
Immunosuppressive regimen (AMTM vs. others)	0.500	0.000, 1.806	0.260	0.526	0.000, 2.818	0.404
Number of DQB1 mismatches (2 vs. 0–1)	0.100	0.000, 0.500	0.025	0.203	0.000, 1.300	0.067

AMTM: Anti-IL-2 receptor antagonists (preoperative) + methylprednisolone (intraoperative) + Tacrolimus + MMF (postoperative).

Bootstrap results were based on 1,000 bootstrap samples.

OR, odd ratio; CI, confidence interval.

**Table 5:** Univariate and multivariate analysis of HLA-DRB1+DQB1 mismatch number and other influencing factors for AR risk in patients who underwent LT using bootstrapping.

Variable	Univariate analysis			Multivariate analysis		
	OR	95% CI	*P*-value	OR	95% CI	*P*-value
Chronic hepatitis B virus infection (Yes vs. NO)	4.200	1.078, 19.708	0.018	3.543	0.624, 42.606	0.108
Operative procedures of liver transplantation (Split vs. Orthotopic)	0.061	0.000, 0.287	0.013	0.092	0.000, 0.497	0.011
Immunosuppressive regimen (AMTM vs. others)	0.500	0.000, 1.806	0.260	0.535	0.000, 2.672	0.419
Number of DRB1+DQB1 mismatches (4 vs. 0–3)	0.200	0.000, 0.811	0.037	0.404	0.000, 2.625	0.172

AMTM: Anti-IL-2 receptor antagonists (preoperative) + methylprednisolone (intraoperative) + tacrolimus + MMF (postoperative).

Bootstrap results were based on 1,000 bootstrap samples.

OR, odd ratio; CI, confidence interval.

## 4 Discussion

AR is a common complication after LT and affects the effectiveness of the transplantation. In this study, the incidence of AR was 13.0%, which was similar to that previously reported by the LT team in our hospital (2) and also in the 10% to 35% approximate range reported by previous studies (12–14). Some studies have reported that recipient age is an independent risk factor for AR after LT (14, 31). However, this phenomenon was not found in our study, which is consistent with the study by Mugaanyi et al. (2). Yu et al. (15) found that HBV infection was associated with AR after LT. This phenomenon was also found in our study, but was not significant after multivariate adjustment. To optimize HBV prophylaxis, antiviral therapy was administered preoperatively to HBV-infected patients, followed by combined antiviral therapy and hepatitis B immunoglobulin (HBIG) postoperatively in our study. Interestingly, we found that split LT was also independently associated with AR, which was not found in other studies (2) and this needs further validation in future studies.

Although HLA is not a routine test before LT, many reports show a correlation between HLA and AR after LT. However, studies on donor-recipient HLA mismatch and AR after LT have reported conflicting conclusions (17, 32–36). Kok et al. (17) found that HLA-C matching reduced the risk of AR after LT, whereas matching at other loci did not reduce AR. A meta-analysis by Lan et al. (32) found that the combination of HLA-A, -B, and -DR loci with low mismatch decreased the risk of AR after LT. A single-center study by Forner et al. (33) found that high HLA-A mismatch increased the risk of AR after LT. However, these associations were not found in the present study, which is consistent with other reports (34–36). Overall, HLA matching has failed to show a consistent advantage for LT, as shown in the review by Reddy et al. (37). The discordant results in this report with other studies may be related to various factors. First, the definition of AR varies across studies, with AR denoting a rejection of the graft within the first 3 months after LT in the present study. Second, the type of LT is inconsistent. Some studies included LDLT or even only LDLT, whereas LDLT was excluded in this study because of the small number of cases. In general, the donor-recipient HLA match is higher in LDLT. Third, the HLA loci and methods of HLA testing were inconsistent. Fourth, confounding variables were erratic, and some studies did not adjust for multiple factors. Finally, HLA itself varies considerably across races and regions.

Immunosuppressants constitute a critical determinant of AR. Therefore, the immunosuppressive regimen was incorporated into the multivariable analysis in our study. However, no statistically significant association was observed, principally attributable to therapeutic homogeneity (>60% of patients received identical protocols in this cohort). Due to the large variation in postoperative dose in the individuals, these immunosuppressant dose differences were excluded from the analysis, which may have biased the results. Although AMR has historically been considered clinically insignificant in LT (38), emerging evidence challenges this paradigm (39, 40). Donor-specific antibodies (DSAs) targeting HLA have been implicated in both acute and chronic liver allograft rejection (39, 40). However, this study was limited to EDTA-anticoagulated specimens and DSAs were not detected, therefore, assessment of their potential role in AR was not conducted.

In renal transplantation, HLA-DQB1 mismatches typically lead to the formation of DSAs, thereby contributing to organ rejection (41, 42). However, as we did not detect DSAs in this study, further studies are needed to verify this observation. Regarding allele-specific effects, higher frequencies of HLA-DQB1*06:01 in the AR donors and -DQB1*05:03 in the non-AR recipients were found but their role needs to be further studied.

In conclusion, this study investigated the correlation between the HLA mismatch between donors and recipients of LT and AR. The association between donor-recipient mismatch and AR in LT at the high-resolution level of 11 HLA loci was investigated by NGS. Although HLA mismatch at the allele level was not an independent risk factor for AR in the multivariate analysis, our data were beneficial for elucidating the relationship between HLA and AR in LT. However, the study has some limitations. First, the sample size was relatively small. The limited sample size resulted in most loci having four or fewer instances of zero mismatches between unrelated donor-recipient pairs. To address potential statistical bias arising from sparse data, we only analyzed combined groups of zero/one mismatches versus two mismatches. Second, as a single-center study, this research may have geographical and population limitations, potentially lacking generalizability and representativeness. Therefore, more multicenter studies with larger sample sizes are warranted to validate the association between HLA and AR after LT.

## 5 Conclusion

In this study, the association between HLA mismatch at the allele level and AR was explored by analyzing the mismatches of a single locus and various combinations of 11 HLA loci in liver transplant donors and recipients at a high-resolution level. However, the available evidence did not support a significant correlation between HLA mismatch and post-transplant AR episodes. Future multicenter cohort studies with larger sample sizes are warranted to validate these findings.

## Data Availability

The raw data supporting the conclusions of this article will be made available by the authors, without undue reservation.

## References

[1] Feng S Roll GR Rouhani FJ Sanchez Fueyo A. The future of liver transplantation. Hepatology. (2024) 80:674–97. doi: 10.1097/HEP.0000000000000873 38537154

[2] Mugaanyi J Tong J Lu C Mao S Huang J Lu C. Risk factors for acute rejection in liver transplantation and its impact on the outcomes of recipients. Transplant Immunol. (2023) 76:101767. doi: 10.1016/j.trim.2022.101767 36470573

[3] Wong YJ Singh N Sanchez-Fernandez N Montano-Loza AJ. Enhanced recovery for liver transplantation: recommendations from the 2022 International Liver Transplantation Society Consensus Conference: towards better clinical care in transplant hepatology. Hepatobiliary Surg Nutr. (2024) 13:512–5. doi: 10.21037/hbsn-24-7 PMC11190513 38911216

[4] Mergental H Laing RW Kirkham AJ Perera M Boteon YL Attard J. Transplantation of discarded livers following viability testing with normothermic machine perfusion. Nat Commun. (2020) 11:2939. doi: 10.1038/s41467-020-16251-3 32546694 PMC7298000

[5] van Leeuwen OB de Vries Y Fujiyoshi M Nijsten MWN Ubbink R Pelgrim GJ. Transplantation of high-risk donor livers after ex situ resuscitation and assessment using combined hypo- and normothermic machine perfusion: A prospective clinical trial. Ann surg. (2019) 270:906–14. doi: 10.1097/SLA.0000000000003540 31633615

[6] Enhanced Recovery after Surgery Committee of Chinese Research Hospital Association. Expert consensus of perioperative management in split liver transplantation(2024). Zhonghua wai ke za zhi [Chinese J surgery]. (2025) 63:1–12. doi: 10.3760/cma.j.cn112139-20240912-00422 39690845

[7] Artru F Sacleux SC Ursic-Bedoya J Ntandja Wandji LC Lutu A L’Hermite S. Long-term outcome following liver transplantation of patients with ACLF grade 3. J hepatol. (2025) 82:62–71. doi: 10.1016/j.jhep.2024.06.039 38981560

[8] Antala S DiNorcia J. Opportunities to improve liver transplantation outcomes in adolescents. J Adolesc Health. (2024) 75:528–9. doi: 10.1016/j.jadohealth.2024.07.002 39293890

[9] Liu Y Pu X Qin X Gong J Huang Z Luo Y. Neutrophil extracellular traps regulate HMGB1 translocation and Kupffer cell M1 polarization during acute liver transplantation rejection. Front Immunol. (2022) 13:823511. doi: 10.3389/fimmu.2022.823511 35603144 PMC9120840

[10] Liang A Zhang L Jia J Zhong K Nie Y. The conclusion of reducing acute rejection after liver transplantation by machine perfusion should be extrapolated with caution. Hepatobiliary Surg Nutr. (2023) 12:785–9. doi: 10.21037/hbsn-23-180 PMC10598305 37886195

[11] Zhang C Chen JZ Dong K Jian YY Huang KY Su RL. Computational identification of novel potential genetic pathogenesis and otherwise biomarkers in acute liver allograft rejection. Heliyon. (2024) 10:e33359. doi: 10.1016/j.heliyon.2024.e33359 39170115 PMC11336371

[12] Soares ME Costa G Guerra L Morais MC Vaz N Codes L. Influence of tacrolimus intrapatient variability on allograft rejection frequency and survival following liver transplantation. Ther Drug monitoring. (2024) 46:456–9. doi: 10.1097/FTD.0000000000001192 38648652

[13] Levitsky J Goldberg D Smith AR Mansfield SA Gillespie BW Merion RM. Acute rejection increases risk of graft failure and death in recent liver transplant recipients. Clin Gastroenterol Hepatol. (2017) 15:584–93.e2. doi: 10.1016/j.cgh.2016.07.035 27567694 PMC5326609

[14] Tang LCY Chetwood JD Lai MSM Yip TCF Cao R Powter E. Incidence, epidemiology, and outcomes of acute allograft rejection following liver transplantation in Australia. Liver Transplant. (2024) 30:1039–49. doi: 10.1097/LVT.0000000000000375 38647419

[15] Yu X Wei B Su R Yao J Feng X Jiang G. A risk assessment model of acute liver allograft rejection by genetic polymorphism of CD276. Mol Genet genomic Med. (2019) 7:e689. doi: 10.1002/mgg3.689 31044564 PMC6603397

[16] Chen R Yi H Zhen J Fan M Xiao L Yu Q. Donor with HLA-C2 is associated with acute rejection following liver transplantation in Southern Chinese. Hla. (2022) 100:133–41. doi: 10.1111/tan.14651 35509131

[17] Kok G Ilcken EF Houwen RHJ Lindemans CA Nieuwenhuis EES Spierings E. The effect of genetic HLA matching on liver transplantation outcome: A systematic review and meta-analysis. Ann Surg Open. (2023) 4:e334. doi: 10.1097/AS9.0000000000000334 37746594 PMC10513352

[18] Robinson J Barker DJ Marsh SGE. 25 years of the IPD-IMGT/HLA database. Hla. (2024) 103:e15549. doi: 10.1111/tan.15549 38936817

[19] Mirza A Ali Qadri MM Zeshan B Hafiz K Abbas S Ahmad N. HLA class-I polymorphisms among the Punjabi population of Pakistan: A comparative analysis with country’s other ethnic groups. Hum Immunol. (2024) 85:111083. doi: 10.1016/j.humimm.2024.111083 39111186

[20] Tao S You X Chen N Dong L Zhao S He Y. The characteristic of HLA-A, HLA-B, HLA-C, HLA-DRB1, HLA-DRB3/4/5, HLA-DQA1, HLA-DQB1, HLA-DPA1, and HLA-DPB1 alleles in Zhejiang Han population. Immunogenetics. (2024) 76:305–14. doi: 10.1007/s00251-024-01349-5 39107575

[21] Mangum DS Caywood E. A clinician’s guide to HLA matching in allogeneic hematopoietic stem cell transplant. Hum Immunol. (2022) 83:687–94. doi: 10.1016/j.humimm.2022.03.002 35346535

[22] Dumortier J Conti F Hiriart JB Dharancy S Duvoux C Besch C. Treatment of donor-specific anti-HLA antibodies-mediated rejection after liver transplantation: A French nationwide retrospective study. Liver Transplant. (2023) 29:1313–22. doi: 10.1097/LVT.0000000000000200 37367954

[23] Wiebe C Nickerson PW. Molecular mismatch-the renaissance of HLA in kidney transplantation. J Am Soc Nephro: JASN. (2020) 31:1922–5. doi: 10.1681/ASN.2020071011 PMC7461661 32868374

[24] Mazzaferro V Regalia E Doci R Andreola S Pulvirenti A Bozzetti F. Liver transplantation for the treatment of small hepatocellular carcinomas in patients with cirrhosis. N Engl J Med. (1996) 334:693–9. doi: 10.1056/NEJM199603143341104 8594428

[25] Xu X Lu D Ling Q Wei X Wu J Zhou L. Liver transplantation for hepatocellular carcinoma beyond the Milan criteria. Gut. (2016) 65:1035–41. doi: 10.1136/gutjnl-2014-308513 PMC4893115 25804634

[26] Wang F Dong L Wang W Chen N Zhang W He J. The polymorphism of HLA-A, -C, -B, -DRB3/4/5, -DRB1, -DQB1 loci in Zhejiang Han population, China using NGS technology. Int J immunogenetics. (2021) 48:485–9. doi: 10.1111/iji.12554 34553840

[27] He Y Li J Mao W Zhang D Liu M Shan X. HLA common and well-documented alleles in China. Hla. (2018) 92:199–205. doi: 10.1111/tan.13358 30073798

[28] Huang J Wang H Fan ST Zhao B Zhang Z Hao L. The national program for deceased organ donation in China. Transplantation. (2013) 96:5–9. doi: 10.1097/TP.0b013e3182985491 23743728

[29] Tajima T Hata K Kusakabe J Miyauchi H Yurugi K Hishida R. The impact of human leukocyte antigen mismatch on recipient outcomes in living-donor liver transplantation. Liver Transplant. (2022) 28:1588–602. doi: 10.1002/lt.26511 PMC9796617 35603526

[30] Excoffier L Lischer HE. Arlequin suite ver 3.5: a new series of programs to perform population genetics analyses under Linux and Windows. Mol Ecol resources. (2010) 10:564–7. doi: 10.1111/j.1755-0998.2010.02847.x 21565059

[31] Wang YC Wu TJ Wu TH Lee CF Chou HS Chan KM. The risk factors to predict acute rejection in liver transplantation. Transplant Proc. (2012) 44:526–8. doi: 10.1016/j.transproceed.2012.01.041 22410062

[32] Lan X Zhang MM Pu CL Guo CB Kang Q Li YC. Impact of human leukocyte antigen mismatching on outcomes of liver transplantation: a meta-analysis. World J gastroenterol. (2010) 16:3457–64. doi: 10.3748/wjg.v16.i27.3457 PMC2904896 20632452

[33] Forner D Liwski R Alwayn I. Human leukocyte antigen, allele, and eplet mismatches in liver transplantation; observations from a small, single center cohort. Hum Immunol. (2018) 79:154–9. doi: 10.1016/j.humimm.2017.12.006 29289739

[34] Kok G Verstegen MMA Houwen RHJ Nieuwenhuis EES Metselaar HJ Polak WG. Assessment of human leukocyte antigen matching algorithm PIRCHE-II on liver transplantation outcomes. Liver Transplant. (2022) 28:1356–66. doi: 10.1002/lt.26431 PMC9544750 35152544

[35] Mittal S Sinha P Sarin S Rastogi A Gupta E Bajpai M. Impact of human leukocyte antigen compatibility on outcomes of living donor liver transplantation: Experience from a tertiary care center. Transplant Infect Dis. (2021) 23:e13644. doi: 10.1111/tid.13644 33999511

[36] Badawy A Kaido T Yoshizawa A Yagi S Fukumitsu K Okajima H. Human leukocyte antigen compatibility and lymphocyte cross-matching play no significant role in the current adult-to-adult living donor liver transplantation. Clin transplantation. (2018) 32:e13234. doi: 10.1111/ctr.13234 29499077

[37] Reddy MS Varghese J Venkataraman J Rela M. Matching donor to recipient in liver transplantation: Relevance in clinical practice. World J hepatol. (2013) 5:603–11. doi: 10.4254/wjh.v5.i11.603 PMC3847943 24303088

[38] Del Bello A Congy-Jolivet N Danjoux M Muscari F Kamar N. Donor-specific antibodies and liver transplantation. Hum Immunol. (2016) 77:1063–70. doi: 10.1016/j.humimm.2016.02.006 26916836

[39] Muro M Legaz I. Importance of human leukocyte antigen antibodies and leukocyte antigen/killer-cell immunoglobulin-like receptor genes in liver transplantation. World J Gastroenterol. (2023) 29:766–72. doi: 10.3748/wjg.v29.i5.766 PMC9932425 36816626

[40] Muro M. The endless history or search for the true role of alloantibodies in liver transplantation. Clin Res Hepatol Gastroenterol. (2021) 45:101544. doi: 10.1016/j.clinre.2020.09.005 33077392

[41] Skulratanasak P Luxsananun T Larpparisuth N Premasathian N Vongwiwatana A. Variations in *de novo* donor-specific antibody development among HLA-DQ mismatches in kidney transplant recipients. PLoS One. (2025) 20:e0321629. doi: 10.1371/journal.pone.0321629 40233060 PMC11999115

[42] Liu W Kang ZY Wang ZL Li DH. Antibody-mediated rejection owing to donor-specific HLA-DQA1 antibodies after renal transplantation: A case report. Transpl Immunol. (2022) 73:101607. doi: 10.1016/j.trim.2022.101607 35477043

