# Supplementary material for: Impact of human leukocyte antigen mismatch between donor-recipient on acute rejection in liver transplantation using next-generation sequencing: a single-center study
Source: Front Immunol. 2025 May 20;16:1576815. doi: 10.3389/fimmu.2025.1576815 (PMC12129760; doi:10.3389/fimmu.2025.1576815)
Supplement: Supplementary file 1 [file DataSheet1.docx]

Table S1. Detailed immunosuppressive regimens.

| Period | Drug type | Specific drug name | Dose | Number |
| --- | --- | --- | --- | --- |
| Preoperative immune induction | Anti-interleukin (IL) -2 receptor antagonist | Basiliximab or Recombinant humanized anti-CD25 monoclonal antibody | 20 mg | 83 |
|  | Unused | - | - | 9 |
| Intraoperative immunosuppression | Glucocorticoid | Methylprednisolone | 500 mg | 92 |
| Postoperative Immunosuppression | Different combinations of calcineurin inhibitors (CNI), antimetabolizers, mammalian target of rapamycin (mTOR), and glucocorticoids | Tacrolimus + Mycophenolate Mofetil (MMF) | Individual adjustment | 63 |
|  |  | Tacrolimus + MMF + Methylprednisolone | Individual adjustment | 5 |
|  |  | Tacrolimus + MMF, Tacrolimus + MMF + Methylprednisolone | Individual adjustment | 6 |
|  |  | Tacrolimus + MMF, Tacrolimus + Myfortic (MPA) | Individual adjustment | 4 |
|  |  | Tacrolimus + MMF, Tacrolimus + Sirolimus | Individual adjustment | 2 |
|  |  | Tacrolimus + MMF, Cyclosporine + MMF | Individual adjustment | 1 |
|  |  | Tacrolimus + MMF, MMF + Sirolimus | Individual adjustment | 2 |
|  |  | Tacrolimus + MMF, Tacrolimus + MMF + Sirolimus | Individual adjustment | 2 |
|  |  | Tacrolimus + MMF, Tacrolimus + MPA | Individual adjustment | 2 |
|  |  | Tacrolimus + MMF + Methylprednisolone, Tacrolimus + Sirolimus + Methylprednisolone | Individual adjustment | 1 |
|  |  | Tacrolimus + MMF + Methylprednisolone, Cyclosporine + MMF | Individual adjustment | 1 |
|  |  | Tacrolimus + MMF, Tacrolimus + Methylprednisolone | Individual adjustment | 1 |
|  |  | Tacrolimus + Methylprednisolone | Individual adjustment | 1 |
|  |  | Tacrolimus + MMF + Methylprednisolone, Cyclosporine + MMF, Cyclosporine + MMF + Methylprednisolone | Individual adjustment | 1 |

Postoperative immunosuppression was observed only at the onset of AR or at 3 months after surgery (without AR).

Table S2. Hardy-Weinberg equilibrium of the 11 HLA loci between liver transplant recipients and donors.

| Locus | Heterozygosity in liver transplant recipients (n=92) | | | Heterozygosity in liver transplant donors (n=71) | | |
| --- | --- | --- | --- | --- | --- | --- |
|  | Observed | Expected | *P* value | Observed | Expected | *P* value |
| HLA-A | 0.82609 | 0.87081 | 0.443 | 0.81690 | 0.84147 | 0.108 |
| HLA-B | 0.92391 | 0.94482 | 0.085 | 0.88732 | 0.92278 | 0.040 |
| HLA-C | 0.82609 | 0.88917 | 0.089 | 0.87324 | 0.89272 | 0.246 |
| HLA-DRB1 | 0.91304 | 0.92195 | 0.057 | 0.94366 | 0.92388 | 0.355 |
| HLA-DRB3 | 0.59783 | 0.59230 | 0.437 | 0.46479 | 0.52772 | 0.303 |
| HLA-DRB4 | 0.53261 | 0.47731 | 0.391 | 0.52113 | 0.42943 | 0.108 |
| HLA-DRB5 | 0.18478 | 0.18110 | 0.356 | 0.35211 | 0.34013 | >0.999 |
| HLA-DQA1 | 0.90217 | 0.90146 | 0.113 | 0.90141 | 0.89422 | 0.135 |
| HLA-DQB1 | 0.89130 | 0.87343 | 0.351 | 0.90141 | 0.88203 | 0.911 |
| HLA-DPA1 | 0.54348 | 0.59094 | 0.014 | 0.57746 | 0.62631 | 0.647 |
| HLA-DPB1 | 0.70652 | 0.73670 | 0.449 | 0.70423 | 0.78933 | 0.118 |

Table S3. Association of the number of mismatches at a single HLA locus and at different combinations of 11 HLA loci with AR via bootstrap analysis.

| HLA loci | Number of mismatches | OR | 95% CI | *P* value |
| --- | --- | --- | --- | --- |
| A | 2 vs. 0-1 | 2.333 | 0.670-13.171 | 0.179 |
| B | 2 vs. 0-1 | 0.642 | 0.000-2.694 | 0.480 |
| C | 2 vs. 0-1 | 2.809 | 0.678-12.528 | 0.085 |
| DRB1 | 2 vs. 0-1 | 0.600 | 0.000-2.321 | 0.420 |
| DRB3 | 2 vs. 0-1 | 1.853 | 0.286-7.078 | 0.307 |
| DRB4 | 2 vs. 0-1 | 0.476 | 0.085-1.893 | 0.244 |
| DRB5 | 2 vs. 0-1 | 1.636 | 0.414-9.747 | 0.442 |
| DQA1 | 2 vs. 0-1 | 0.496 | 0.000-1.994 | 0.289 |
| DQB1 | 2 vs. 0-1 | 0.100 | 0.000-0.500 | 0.025 |
| DPA1 | 2 vs. 0-1 | 0.379 | 0.085-1.586 | 0.123 |
| DPB1 | 2 vs. 0-1 | 0.905 | 0.238-3.248 | 0.876 |
| A+B+C | 4-6 vs 2-3 | 0.818 | 0.000-4.437 | 0.501 |
| A+B+C | 6 vs 2-5 | 1.556 | 0.404-12.949 | 0.481 |
| A+B+DRB1 | 4-6 vs 2-3 | 1.121 | 0.000-6.994 | 0.568 |
| A+B+DRB1 | 6 vs 2-5 | 1.405 | 0.365-8.551 | 0.608 |
| DRB1+DQB1 | 4 vs 0-3 | 0.200 | 0.000-0.811 | 0.037 |
| A+B+C+DRB1+DQB1 | 8-10 vs 4-7 | 0.879 | 0.000-3.401 | 0.818 |
| All Class II | 9-13 vs 4-8 | 0.316 | 0.000-1.182 | 0.089 |
| All Class II | 11-13 vs 4-10 | 0.481 | 0.101-2.008 | 0.242 |
| 11 loci | 12-19 vs 8-11 | 0.515 | 0.000-2.469 | 0.268 |
| 11 loci | 17-19 vs 8-16 | 0.540 | 0.150-3.508 | 0.333 |

Bootstrap results were based on 1000 bootstrap samples.

Table S4. Comparison of the mismatch of a single locus and various combinations of 11 HLA loci between AR and non-AR groups.

| HLA loci | Number of mismatches | AR (n=12) | Non-AR (n=80) | *P* value |
| --- | --- | --- | --- | --- |
| A | 0 | 1 (8.3) | 3 (3.8) | 0.263 |
|  | 1 | 6 (50) | 27 (33.8) |  |
|  | 2 | 5 (41.7) | 50 (62.5) |  |
| B | 0 | 0 (0.0) | 0 (0.0) | 0.860 |
|  | 1 | 2 (16.7) | 19 (23.8) |  |
|  | 2 | 10 (83.3) | 61 (76.3) |  |
| C | 0 | 0 (0.0) | 2 (2.5) | 0.222 |
|  | 1 | 6 (50.0) | 19 (23.8) |  |
|  | 2 | 6 (50.0) | 59 (73.8) |  |
| DRB1 | 0 | 0 (0.0) | 1 (1.3) | 0.739 |
|  | 1 | 2 (16.7) | 19 (23.8) |  |
|  | 2 | 10 (83.3) | 60 (75.0) |  |
| DRB3 | 0 | 4 (33.3) | 17 (21.3) | 0.592 |
|  | 1 | 6 (50.0) | 52 (65.0) |  |
|  | 2 | 2 (16.7) | 11 (13.8) |  |
| DRB4 | 0 | 4 (33.3) | 41 (51.3) | 0.423 |
|  | 1 | 8 (66.7) | 36 (45.0) |  |
|  | 2 | 0 (0.0) | 3 (3.8) |  |
| DRB5 | 0 | 8 (66.7) | 44 (55.0) | 0.839 |
|  | 1 | 4 (33.3) | 33 (41.3) |  |
|  | 2 | 0 (0.0) | 3 (3.8) |  |
| DQA1 | 0 | 0 (0.0) | 3 (3.8) | 0.819 |
|  | 1 | 2 (16.7) | 20 (25.0) |  |
|  | 2 | 10 (83.3) | 57 (71.3) |  |
| DQB1 | 0 | 0 (0.0) | 4 (5.0) | 0.034 |
|  | 1 | 1 (8.3) | 34 (42.5) |  |
|  | 2 | 11 (91.7) | 42 (52.5) |  |
| DPA1 | 0 | 1 (8.3) | 16 (20.0) | 0.346 |
|  | 1 | 5 (41.7) | 42 (52.5) |  |
|  | 2 | 6 (50.0) | 22 (27.5) |  |
| DPB1 | 0 | 0 (0.0) | 10 (12.5) | 0.610 |
|  | 1 | 6 (50.0) | 32 (40.0) |  |
|  | 2 | 6 (50.0) | 38 (47.5) |  |
| A+B+C | 2 | 1 (8.3) | 2 (2.5) | 0.138 |
|  | 3 | 0 (0.0) | 6 (7.5) |  |
|  | 4 | 5 (41.7) | 12 (15.0) |  |
|  | 5 | 2 (16.7) | 25 (31.3) |  |
|  | 6 | 4 (33.3) | 35 (43.8) |  |
| A+B+DRB1 | 2 | 0 (0.0) | 1 (1.3) | 0.912 |
|  | 3 | 1 (8.3) | 5 (6.3) |  |
|  | 4 | 2 (16.7) | 13 (16.3) |  |
|  | 5 | 5 (41.7) | 28 (35.0) |  |
|  | 6 | 4 (33.3) | 33 (41.3) |  |
| DRB1+DQB1 | 0 | 0 (0.0) | 1 (1.3) | 0.337 |
|  | 1 | 0 (0.0) | 2 (2.5) |  |
|  | 2 | 1 (8.3) | 16 (20.0) |  |
|  | 3 | 1 (8.3) | 21 (26.3) |  |
|  | 4 | 10 (83.3) | 40 (50.0) |  |
| A+B+C+DRB1+DQB1 | 4 | 0 (0.0) | 1 (1.3) | 0.989 |
|  | 5 | 0 (0.0) | 4 (5.0) |  |
|  | 6 | 2 (16.7) | 8 (10.0) |  |
|  | 7 | 1 (8.3) | 9 (11.3) |  |
|  | 8 | 3 (25.0) | 18 (22.5) |  |
|  | 9 | 2 (16.7) | 17 (21.3) |  |
|  | 10 | 4 (33.3) | 23 (28.8) |  |
| All Class II | 4 | 0 (0.0) | 1 (1.3) | 0.720 |
|  | 5 | 0 (0.0) | 3 (3.8) |  |
|  | 6 | 0 (0.0) | 2 (2.5) |  |
|  | 7 | 1 (8.3) | 13 (16.3) |  |
|  | 8 | 1 (8.3) | 12 (15.0) |  |
|  | 9 | 3 (25.0) | 10 (12.5) |  |
|  | 10 | 1 (8.3) | 13 (16.3) |  |
|  | 11 | 2 (16.7) | 13 (16.3) |  |
|  | 12 | 2 (16.7) | 10 (12.5) |  |
|  | 13 | 2 (16.7) | 3 (3.8) |  |
| 11 loci | 8 | 0 (0.0) | 1 (1.3) | 0.514 |
|  | 9 | 0 (0.0) | 1 (1.3) |  |
|  | 10 | 0 (0.0) | 6 (7.5) |  |
|  | 11 | 1 (8.3) | 4 (5.0) |  |
|  | 12 | 0 (0.0) | 6 (7.5) |  |
|  | 13 | 4 (33.3) | 12 (15.0) |  |
|  | 14 | 1 (8.3) | 9 (11.3) |  |
|  | 15 | 0 (0.0) | 13 (16.3) |  |
|  | 16 | 2 (16.7) | 11 (13.8) |  |
|  | 17 | 2 (16.7) | 8 (10.0) |  |
|  | 18 | 1 (8.3) | 8 (10.0) |  |
|  | 19 | 1 (8.3) | 1 (1.3) |  |

Table S5. Characteristics of two HLA-DQB1 mismatch types in donor-recipient pairs of 11 AR cases.

| Recipients (n=11) | | Donors (n=11) | |
| --- | --- | --- | --- |
| DQB1*02:01 | DQB1*02:02 | DQB1*06:01 | DQB1*06:01 |
| DQB1*02:01 | DQB1*02:01 | DQB1*05:03 | DQB1*06:01 |
| DQB1*03:01 | DQB1*05:02 | DQB1*06:01 | DQB1*06:02 |
| DQB1*03:01 | DQB1*05:02 | DQB1*03:02 | DQB1*06:01 |
| DQB1*03:01 | DQB1*06:01 | DQB1*03:03 | DQB1*05:01 |
| DQB1*03:02 | DQB1*06:01 | DQB1*03:03 | DQB1*05:03 |
| DQB1*03:03 | DQB1*05:01 | DQB1*06:01 | DQB1*06:01 |
| DQB1*03:03 | DQB1*05:02 | DQB1*02:01 | DQB1*03:02 |
| DQB1*03:03 | DQB1*05:03 | DQB1*03:01 | DQB1*06:01 |
| DQB1*03:03 | DQB1*04:01 | DQB1*03:01 | DQB1*06:01 |
| DQB1*05:03 | DQB1*06:01 | DQB1*02:02 | DQB1*03:01 |

Table S6. Characteristics of two HLA-DQB1 mismatch types in donor-recipient pairs of 42 non-AR cases.

| Recipients (n=42) | | Donors (n=42) | |
| --- | --- | --- | --- |
| DQB1*02:01 | DQB1*02:01 | DQB1*03:03 | DQB1*05:02 |
| DQB1*02:01 | DQB1*03:03 | DQB1*03:01 | DQB1*03:02 |
| DQB1*02:01 | DQB1*03:03 | DQB1*03:02 | DQB1*04:02 |
| DQB1*02:01 | DQB1*03:03 | DQB1*06:01 | DQB1*06:01 |
| DQB1*02:01 | DQB1*05:02 | DQB1*03:01 | DQB1*05:01 |
| DQB1*02:02 | DQB1*03:01 | DQB1*03:02 | DQB1*06:01 |
| DQB1*02:02 | DQB1*03:01 | DQB1*05:02 | DQB1*06:01 |
| DQB1*02:02 | DQB1*03:03 | DQB1*02:01 | DQB1*03:02 |
| DQB1*02:02 | DQB1*03:03 | DQB1*03:01 | DQB1*06:01 |
| DQB1*02:02 | DQB1*05:02 | DQB1*03:03 | DQB1*05:01 |
| DQB1*02:02 | DQB1*05:03 | DQB1*05:02 | DQB1*06:01 |
| DQB1*02:02 | DQB1*06:01 | DQB1*03:01 | DQB1*06:09 |
| DQB1*02:02 | DQB1*06:02 | DQB1*03:03 | DQB1*06:01 |
| DQB1*02:02 | DQB1*06:04 | DQB1*03:03 | DQB1*03:03 |
| DQB1*03:01 | DQB1*03:01 | DQB1*03:03 | DQB1*06:02 |
| DQB1*03:01 | DQB1*03:01 | DQB1*03:03 | DQB1*06:09 |
| DQB1*03:01 | DQB1*03:01 | DQB1*05:01 | DQB1*06:02 |
| DQB1*03:01 | DQB1*03:03 | DQB1*06:01 | DQB1*06:02 |
| DQB1*03:01 | DQB1*04:01 | DQB1*03:02 | DQB1*06:02 |
| DQB1*03:01 | DQB1*05:02 | DQB1*02:02 | DQB1*03:03 |
| DQB1*03:01 | DQB1*05:03 | DQB1*03:03 | DQB1*05:01 |
| DQB1*03:01 | DQB1*06:01 | DQB1*05:02 | DQB1*05:03 |
| DQB1*03:01 | DQB1*06:02 | DQB1*05:02 | DQB1*05:03 |
| DQB1*03:01 | DQB1*06:02 | DQB1*06:01 | DQB1*06:09 |
| DQB1*03:02 | DQB1*03:03 | DQB1*04:01 | DQB1*05:01 |
| DQB1*03:02 | DQB1*03:03 | DQB1*06:01 | DQB1*06:01 |
| DQB1*03:02 | DQB1*06:02 | DQB1*03:03 | DQB1*05:03 |
| DQB1*03:03 | DQB1*03:03 | DQB1*03:02 | DQB1*06:02 |
| DQB1*03:03 | DQB1*04:02 | DQB1*04:01 | DQB1*05:03 |
| DQB1*03:03 | DQB1*05:03 | DQB1*02:01 | DQB1*02:02 |
| DQB1*03:03 | DQB1*06:01 | DQB1*02:01 | DQB1*05:02 |
| DQB1*03:03 | DQB1*06:01 | DQB1*03:01 | DQB1*03:02 |
| DQB1*03:03 | DQB1*06:01 | DQB1*04:01 | DQB1*05:03 |
| DQB1*04:01 | DQB1*05:03 | DQB1*02:02 | DQB1*05:02 |
| DQB1*04:01 | DQB1*05:03 | DQB1*03:01 | DQB1*03:01 |
| DQB1*04:01 | DQB1*06:02 | DQB1*03:02 | DQB1*04:02 |
| DQB1*04:02 | DQB1*05:03 | DQB1*03:02 | DQB1*06:02 |
| DQB1*05:02 | DQB1*05:02 | DQB1*03:01 | DQB1*03:01 |
| DQB1*05:02 | DQB1*05:03 | DQB1*02:01 | DQB1*03:01 |
| DQB1*05:02 | DQB1*05:03 | DQB1*03:01 | DQB1*06:01 |
| DQB1*05:02 | DQB1*06:01 | DQB1*03:01 | DQB1*05:01 |
| DQB1*06:04 | DQB1*06:09 | DQB1*03:01 | DQB1*03:03 |

Table S7. Comparison of HLA-DQB1 alleles between AR and non-AR recipient groups under HLA-DQB1 two-alleles mismatch in donor-recipient pairs.

| HLA allele | AR recipients (N=11) | Non-AR recipients (N=42) | OR | 95% CI for OR | *P* value | *Pc* value |
| --- | --- | --- | --- | --- | --- | --- |
|  | % | % |  |  |  |  |
| DQB1*02:01 | 13.64 | 7.14 | 2.053 | 0.470-8.962 | 0.587 | >0.999 |
| DQB1*02:02 | 4.55 | 10.71 | 0.397 | 0.048-3.312 | 0.637 | >0.999 |
| DQB1*03:01 | 13.64 | 17.86 | 0.726 | 0.190-2.773 | 0.880 | >0.999 |
| DQB1*03:02 | 4.55 | 3.57 | 1.286 | 0.127-12.998 | >0.999 | >0.999 |
| DQB1*03:03 | 18.18 | 17.86 | 1.022 | 0.302-3.458 | >0.999 | >0.999 |
| DQB1*04:01 | 4.55 | 4.76 | 0.95 | 0.101-8.976 | >0.999 | >0.999 |
| DQB1*04:02 | 0.00 | 2.38 | - | - | >0.999 | >0.999 |
| DQB1*05:01 | 4.55 | 0.00 | - | - | 0.208 | >0.999 |
| DQB1*05:02 | 13.64 | 9.52 | 1.500 | 0.363-6.199 | 0.865 | >0.999 |
| DQB1*05:03 | 9.09 | 9.52 | 0.950 | 0.187-4.829 | >0.999 | >0.999 |
| DQB1*06:01 | 13.64 | 7.14 | 2.053 | 0.470-8.962 | 0.587 | >0.999 |
| DQB1*06:02 | 0.00 | 5.95 | - | - | 0.581 | >0.999 |
| DQB1*06:04 | 0.00 | 2.38 | - | - | >0.999 | >0.999 |
| DQB1*06:09 | 0.00 | 1.19 | - | - | >0.999 | >0.999 |

The *P* values of allele frequencies (AFs) were corrected by the step-down Bonferroni method (*Pc* values).

Table S8. Comparison of HLA-DQB1 alleles between AR recipients and Zhejiang Han healthy controls under donor-recipient HLA-DQB1 two-alleles mismatch.

| HLA allele | AR recipients (N=11) | Controls (N=813) | OR | 95% CI for OR | *P* value | *Pc* value |
| --- | --- | --- | --- | --- | --- | --- |
|  | % | % |  |  |  |  |
| DQB1*02:01 | 13.64 | 6.21 | 2.384 | 0.694-8.191 | 0.326 | >0.999 |
| DQB1*02:02 | 4.55 | 4.43 | 1.028 | 0.136-7.747 | >0.999 | >0.999 |
| DQB1*03:01 | 13.64 | 21.71 | 0.569 | 0.168-1.935 | 0.514 | >0.999 |
| DQB1*03:02 | 4.55 | 6.83 | 0.650 | 0.087-4.877 | >0.999 | >0.999 |
| DQB1*03:03 | 18.18 | 18.64 | 0.970 | 0.326-2.888 | >0.999 | >0.999 |
| DQB1*03:13 | 0.00 | 0.06 | - | - | >0.999 | >0.999 |
| DQB1*03:158 | 0.00 | 0.06 | - | - | >0.999 | >0.999 |
| DQB1*04:01 | 4.55 | 5.90 | 0.759 | 0.101-5.702 | >0.999 | >0.999 |
| DQB1*04:02 | 0.00 | 1.42 | - | - | >0.999 | >0.999 |
| DQB1*05:01 | 4.55 | 4.31 | 1.059 | 0.140-7.982 | >0.999 | >0.999 |
| DQB1*05:02 | 13.64 | 6.40 | 2.311 | 0.673-7.935 | 0.351 | >0.999 |
| DQB1*05:03 | 9.09 | 2.77 | 3.513 | 0.797-15.487 | 0.128 | >0.999 |
| DQB1*06:01 | 13.64 | 11.69 | 1.193 | 0.350-4.071 | >0.999 | >0.999 |
| DQB1*06:02 | 0.00 | 6.03 | - | - | 0.463 | >0.999 |
| DQB1*06:02:01G | 0.00 | 0.06 | - | - | >0.999 | >0.999 |
| DQB1*06:03 | 0.00 | 0.62 | - | - | >0.999 | >0.999 |
| DQB1*06:04 | 0.00 | 0.55 | - | - | >0.999 | >0.999 |
| DQB1*06:09 | 0.00 | 2.09 | - | - | >0.999 | >0.999 |
| DQB1*06:09:01G | 0.00 | 0.06 | - | - | >0.999 | >0.999 |
| DQB1*06:10 | 0.00 | 0.25 | - | - | >0.999 | >0.999 |
| DQB1*06:108 | 0.00 | 0.06 | - | - | >0.999 | >0.999 |

The *P* values of AFs were corrected by the step-down Bonferroni method (*Pc* values).

Table S9. Comparison of HLA-DQB1 alleles between non-AR recipients and Zhejiang Han healthy controls under donor-recipient HLA-DQB1 two-allele mismatch.

| HLA allele | Non-AR recipients (N=42) | Controls (N=813) | OR | 95% CI for OR | *P* value | *Pc* value |
| --- | --- | --- | --- | --- | --- | --- |
|  | % | % |  |  |  |  |
| DQB1*02:01 | 7.14 | 6.21 | 1.161 | 0.494-2.729 | 0.731 | >0.999 |
| DQB1*02:02 | 10.71 | 4.43 | 2.590 | 1.247-5.378 | 0.017 | 0.340 |
| DQB1*03:01 | 17.86 | 21.71 | 0.784 | 0.443-1.387 | 0.402 | >0.999 |
| DQB1*03:02 | 3.57 | 6.83 | 0.506 | 0.157-1.626 | 0.244 | >0.999 |
| DQB1*03:03 | 17.86 | 18.64 | 0.949 | 0.536-1.682 | 0.858 | >0.999 |
| DQB1*03:13 | 0.00 | 0.06 | - | - | >0.999 | >0.999 |
| DQB1*03:158 | 0.00 | 0.06 | - | - | >0.999 | >0.999 |
| DQB1*04:01 | 4.76 | 5.90 | 0.797 | 0.286-2.221 | 0.844 | >0.999 |
| DQB1*04:02 | 2.38 | 1.42 | 1.700 | 0.394-7.333 | 0.800 | >0.999 |
| DQB1*05:01 | 0.00 | 4.31 | - | - | 0.097 | >0.999 |
| DQB1*05:02 | 9.52 | 6.40 | 1.540 | 0.724-3.278 | 0.259 | >0.999 |
| DQB1*05:03 | 9.52 | 2.77 | 3.698 | 1.684-8.120 | <0.001 | 0.010 |
| DQB1*06:01 | 7.14 | 11.69 | 0.581 | 0.250-1.352 | 0.203 | >0.999 |
| DQB1*06:02 | 5.95 | 6.03 | 0.987 | 0.391-2.493 | 0.978 | >0.999 |
| DQB1*06:02:01G | 0.00 | 0.06 | - | - | >0.999 | >0.999 |
| DQB1*06:03 | 0.00 | 0.62 | - | - | >0.999 | >0.999 |
| DQB1*06:04 | 2.38 | 0.55 | 4.382 | 0.932-20.608 | 0.098 | >0.999 |
| DQB1*06:09 | 1.19 | 2.09 | 0.564 | 0.076-4.172 | 0.862 | >0.999 |
| DQB1*06:09:01G | 0.00 | 0.06 | - | - | >0.999 | >0.999 |
| DQB1*06:10 | 0.00 | 0.25 | - | - | >0.999 | >0.999 |
| DQB1*06:108 | 0.00 | 0.06 | - | - | >0.999 | >0.999 |

The *P* values of AFs were corrected by the step-down Bonferroni method (*Pc* values).

Table S10. Comparison of HLA-DQB1 alleles between AR and non-AR donor groups under HLA-DQB1 two-alleles mismatch in donor-recipient pairs.

| HLA allele | AR donors (N=11) | Non-AR donors (N=42) | OR | 95% CI for OR | *P* value | *Pc* value |
| --- | --- | --- | --- | --- | --- | --- |
|  | % | % |  |  |  |  |
| DQB1*02:01 | 4.55 | 4.76 | 0.952 | 0.101-8.976 | >0.999 | >0.999 |
| DQB1*02:02 | 4.55 | 3.57 | 1.286 | 0.127-12.998 | >0.999 | >0.999 |
| DQB1*03:01 | 13.64 | 15.48 | 0.862 | 0.223-3.338 | >0.999 | >0.999 |
| DQB1*03:02 | 9.09 | 10.71 | 0.883 | 0.167-4.167 | >0.999 | >0.999 |
| DQB1*03:03 | 9.09 | 13.10 | 0.664 | 0.136-3.241 | 0.885 | >0.999 |
| DQB1*04:01 | 0.00 | 3.57 | - | - | >0.999 | >0.999 |
| DQB1*04:02 | 0.00 | 2.38 | - | - | >0.999 | >0.999 |
| DQB1*05:01 | 4.55 | 7.14 | 0.619 | 0.071-5.428 | >0.999 | >0.999 |
| DQB1*05:02 | 0.00 | 8.33 | - | - | 0.358 | >0.999 |
| DQB1*05:03 | 9.09 | 5.95 | 1.580 | 0.285-8.750 | 0.964 | >0.999 |
| DQB1*06:01 | 40.91 | 14.29 | 4.154 | 1.458-11.832 | 0.013 | 0.169 |
| DQB1*06:02 | 4.55 | 7.14 | 0.619 | 0.071-5.428 | >0.999 | >0.999 |
| DQB1*06:09 | 0.00 | 3.57 | - | - | >0.999 | >0.999 |

The *P* values of AFs were corrected by the step-down Bonferroni method (*Pc* values).

Table S11. Comparison of HLA-DQB1 alleles between AR donors and Zhejiang Han healthy controls under donor-recipient HLA-DQB1 two-alleles mismatch.

| HLA allele | AR donors (N=11) | Controls (N=813) | OR | 95% CI for OR | *P* value | *Pc* value |
| --- | --- | --- | --- | --- | --- | --- |
|  | % | % |  |  |  |  |
| DQB1*02:01 | 4.55 | 6.21 | 0.719 | 0.096-5.399 | >0.999 | >0.999 |
| DQB1*02:02 | 4.55 | 4.43 | 1.028 | 0.136-7.747 | >0.999 | >0.999 |
| DQB1*03:01 | 13.64 | 21.71 | 0.569 | 0.168-1.935 | 0.514 | >0.999 |
| DQB1*03:02 | 9.09 | 6.83 | 1.365 | 0.315-5.914 | >0.999 | >0.999 |
| DQB1*03:03 | 9.09 | 18.64 | 0.437 | 0.102-1.878 | 0.385 | >0.999 |
| DQB1*03:13 | 0.00 | 0.06 | - | - | >0.999 | >0.999 |
| DQB1*03:158 | 0.00 | 0.06 | - | - | >0.999 | >0.999 |
| DQB1*04:01 | 0.00 | 5.90 | - | - | 0.474 | >0.999 |
| DQB1*04:02 | 0.00 | 1.42 | - | - | >0.999 | >0.999 |
| DQB1*05:01 | 4.55 | 4.31 | 1.059 | 0.140-7.982 | >0.999 | >0.999 |
| DQB1*05:02 | 0.00 | 6.40 | - | - | 0.433 | >0.999 |
| DQB1*05:03 | 9.09 | 2.77 | 3.513 | 0.797-15.487 | 0.128 | >0.999 |
| DQB1*06:01 | 40.91 | 11.69 | 5.232 | 2.207-12.405 | <0.001 | 0.002 |
| DQB1*06:02 | 4.55 | 6.03 | 0.742 | 0.099-5.577 | >0.999 | >0.999 |
| DQB1*06:02:01G | 0.00 | 0.06 | - | - | >0.999 | >0.999 |
| DQB1*06:03 | 0.00 | 0.62 | - | - | >0.999 | >0.999 |
| DQB1*06:04 | 0.00 | 0.55 | - | - | >0.999 | >0.999 |
| DQB1*06:09 | 0.00 | 2.09 | - | - | >0.999 | >0.999 |
| DQB1*06:09:01G | 0.00 | 0.06 | - | - | >0.999 | >0.999 |
| DQB1*06:10 | 0.00 | 0.25 | - | - | >0.999 | >0.999 |
| DQB1*06:108 | 0.00 | 0.06 | - | - | >0.999 | >0.999 |

The *P* values of AFs were corrected by the step-down Bonferroni method (*Pc* values).

Table S12. Comparison of HLA-DQB1 alleles between non-AR donors and Zhejiang Han healthy controls under donor-recipient HLA-DQB1 two-alleles mismatch.

| HLA allele | Non-AR donors (N=42) | Controls (N=813) | OR | 95% CI for OR | *P* value | *Pc* value |
| --- | --- | --- | --- | --- | --- | --- |
|  | % | % |  |  |  |  |
| DQB1*02:01 | 4.76 | 6.21 | 0.755 | 0.271-2.102 | 0.589 | >0.999 |
| DQB1*02:02 | 3.57 | 4.43 | 0.789 | 0.247-2.592 | 0.920 | >0.999 |
| DQB1*03:01 | 15.48 | 21.71 | 0.660 | 0.361-1.207 | 0.174 | >0.999 |
| DQB1*03:02 | 10.71 | 6.83 | 1.638 | 0.799-3.357 | 0.174 | >0.999 |
| DQB1*03:03 | 13.10 | 18.64 | 0.658 | 0.345-1.255 | 0.201 | >0.999 |
| DQB1*03:13 | 0.00 | 0.06 | - | - | >0.999 | >0.999 |
| DQB1*03:158 | 0.00 | 0.06 | - | - | >0.999 | >0.999 |
| DQB1*04:01 | 3.57 | 5.90 | 0.590 | 0.183-1.903 | 0.514 | >0.999 |
| DQB1*04:02 | 2.38 | 1.42 | 1.700 | 0.394-7.333 | 0.800 | >0.999 |
| DQB1*05:01 | 7.14 | 4.31 | 1.710 | 0.721-4.058 | 0.337 | >0.999 |
| DQB1*05:02 | 8.33 | 6.40 | 1.330 | 0.598-2.957 | 0.482 | >0.999 |
| DQB1*05:03 | 5.95 | 2.77 | 2.224 | 0.859-5.756 | 0.175 | >0.999 |
| DQB1*06:01 | 14.29 | 11.69 | 1.260 | 0.671-2.364 | 0.471 | >0.999 |
| DQB1*06:02 | 7.14 | 6.03 | 1.199 | 0.510-2.820 | 0.676 | >0.999 |
| DQB1*06:02:01G | 0.00 | 0.06 | - | - | >0.999 | >0.999 |
| DQB1*06:03 | 0.00 | 0.62 | - | - | >0.999 | >0.999 |
| DQB1*06:04 | 0.00 | 0.55 | - | - | >0.999 | >0.999 |
| DQB1*06:09 | 3.57 | 2.09 | 1.734 | 0.522-5.766 | 0.600 | >0.999 |
| DQB1*06:09:01G | 0.00 | 0.06 | - | - | >0.999 | >0.999 |
| DQB1*06:10 | 0.00 | 0.25 | - | - | >0.999 | >0.999 |
| DQB1*06:108 | 0.00 | 0.06 | - | - | >0.999 | >0.999 |

The *P* values of AFs were corrected by the step-down Bonferroni method (*Pc* values).

Table S13. Compare the number of HLA mismatches between the AR and non-AR groups, including mismatches of 11 HLA loci and mismatches of various loci combinations, when the HLA mismatch was counted as the number of donor HLA that the recipient does not have.

| HLA loci | Number of mismatches | AR (n=12) | Non-AR (n=80) | *P* value |
| --- | --- | --- | --- | --- |
| A | 0-1 | 7 (58.3) | 39 (48.8) | 0.536 |
|  | 2 | 5 (41.7) | 41 (51.3) |  |
| B | 0-1 | 2 (16.9) | 26 (32.5) | 0.438 |
|  | 2 | 10 (83.3) | 54 (67.5) |  |
| C | 0-1 | 6 (50.0) | 29 (36.3) | 0.551 |
|  | 2 | 6 (50.0) | 51 (63.8) |  |
| DRB1 | 0-1 | 3 (25.0) | 24 (30.0) | 0.988 |
|  | 2 | 9 (75.0) | 56 (70.0) |  |
| DRB3 | 0 | 6 (50.0) | 38 (47.5) | 0.872 |
|  | 1-2 | 6 (50.0) | 42 (52.5) |  |
| DRB4 | 0 | 8 (66.7) | 58 (72.5) | 0.940 |
|  | 1 | 4 (33.3) | 22 (27.5) |  |
| DRB5 | 0 | 10 (83.3) | 50 (62.5) | 0.277 |
|  | 1 | 2 (16.7) | 30 (37.5) |  |
| DQA1 | 0-1 | 3 (25.0) | 28 (35.0) | 0.722 |
|  | 2 | 9 (75.0) | 52 (65.0) |  |
| DQB1 | 0-1 | 3 (25.0) | 43 (53.8) | 0.063 |
|  | 2 | 9 (75.0) | 37 (46.3) |  |
| DPA1 | 0-1 | 8 (66.7) | 71 (88.8) | 0.109 |
|  | 2 | 4 (33.3) | 9 (11.3) |  |
| DPB1 | 0-1 | 8 (66.7) | 53 (66.3) | 1.000 |
|  | 2 | 4 (33.3) | 27 (33.8) |  |
| A+B+C | 2-3 | 1 (8.3) | 16 (20.0) | 0.567 |
|  | 4-6 | 11 (91.7) | 64 (80.0) |  |
| A+B+C | 2-5 | 8 (66.7) | 54 (67.5) | 1.000 |
|  | 6 | 4 (33.3) | 26 (32.5) |  |
| A+B+DRB1 | 2-3 | 1 (8.3) | 13 (16.3) | 0.779 |
|  | 4-6 | 11 (91.7) | 67 (83.8) |  |
| A+B+DRB1 | 2-5 | 8 (66.7) | 55 (68.8) | 1.000 |
|  | 6 | 4 (33.3) | 25 (31.3) |  |
| DRB1+DQB1 | 0-2 | 2 (16.7) | 23 (28.8) | 0.596 |
|  | 3-4 | 10 (83.3) | 57 (71.3) |  |
| DRB1+DQB1 | 0-3 | 4 (33.3) | 45 (56.3) | 0.138 |
|  | 4 | 8 (66.7) | 35 (43.8) |  |
| A+B+C+DRB1+DQB1 | 4-5 | 0 (0.0) | 9 (11.3) | 0.483 |
|  | 6-10 | 12 (100.0) | 71 (88.8) |  |
| A+B+C+DRB1+DQB1 | 4-7 | 4 (33.3) | 35 (43.8) | 0.496 |
|  | 8-10 | 8 (66.7) | 45 (56.3) |  |
| All Class II | 4-8 | 6 (50.0) | 53 (66.3) | 0.440 |
|  | 9-11 | 6 (50.0) | 27 (33.8) |  |
| 11 loci | 7-11 | 4 (33.3) | 31 (38.8) | 0.967 |
|  | 12-17 | 8 (66.7) | 49 (61.3) |  |
| 11 loci | 7-14 | 6 (50.0) | 62 (77.5) | 0.095 |
|  | 15-17 | 6 (50.0) | 18 (22.5) |  |
